# Determinants of public trust in complementary and alternative medicine

**DOI:** 10.1186/1471-2458-10-128

**Published:** 2010-03-12

**Authors:** Evelien van der Schee, Peter P Groenewegen

**Affiliations:** 1 ParnassiaBavogroep, Brijder Addiction Care, Parnassia Addiction Research Center, The Hague, Monsterseweg 83, 2553 RJ, The Netherlands; 2 NIVEL -Netherlands Institute for Health Services Research, Utrecht, The Netherlands; 3 Utrecht University, Department of Sociology, Department of Human Geography, Utrecht, The Netherlands

## Abstract

**Background:**

In the Netherlands, public trust in conventional medicine is relatively high. There is reason to believe that public trust in complementary and alternative medicine (CAM) is rated lower. The aim of this study is to gain insight into public trust in CAM and the determinants that lie at the root of it. We hypothesized that public trust in CAM is related to (perceived) institutional guarantees, media information on CAM, information from people's social network, personal experiences, the role of general practitioners (GPs) and trust in conventional medicine.

**Methods:**

A postal questionnaire on public trust in CAM was mailed to 1358 members of the Health Care Consumer Panel. 65% of the questionnaires were returned. Data were analysed using frequencies, ANOVA, post hoc testing and linear regression analyses.

**Results:**

In the total sample, the level of public trust in CAM was a 5.05 on average on a scale of 1-10. 40.7% was CAM user (current or past) and displayed significantly higher levels of trust toward CAM than CAM non users. In the total sample, public trust in CAM was related to institutional guarantees, negative media information, positive and negative information reported by their social network and people's personal experiences with CAM. For non users, trust is mostly associated with institutional guarantees. For users, personal experiences are most important. For both users and non users, trust levels in CAM are affected by negative media information. Public trust in CAM is for CAM users related to positive information and for non users to negative information from their network.

**Conclusions:**

In the Netherlands, CAM is trusted less than conventional medicine. The hypotheses on institutional guarantees, media information, information from the network and people's personal experiences are confirmed by our study for the total sample, CAM non users and users. The other hypotheses are rejected.

## Background

For lay people, the use of health care involves risk taking. Risk taking is inherent to health care as for patients it is difficult to judge whether they received the right type of care, the right amount of care or good quality care. In other words, there is information asymmetry between patients as principals and health care providers as their agents [1-3]. Patients have to rely on the expert knowledge of their caregivers and to *trust *that their caregivers are providing them with good quality care [4-6]. This means health care is a confidence good; a good in which trust is an important mechanism.

In the field of health care trust is generally understood as defined by Hall et al [7]: "the optimistic acceptance of a vulnerable situation in which the truster believes the trustee will care for the truster's interests." Two types of trust can be distinguished: interpersonal and public trust. Interpersonal trust is trust placed by one person in another. This corresponds to the description of Hall and others. Public trust is trust placed by a group or a person in a societal institution or system, also described as "being confident that you will be adequately treated when you are in need of health care" [8]. It reflects a general assumption about whether a system or institution can be trusted. Public trust is expected to be useful as a performance indicator of the health care system and professions working in it. In the Dutch Health Care Performance Report [9], for instance, one of the health care indicators is public trust.

Dutch people rate their level of public trust in the health care system with 7 on a scale from 1(no trust) to10 (high trust) [10]. Health care professions are highly trusted. Rated on a 1 to 4 scale from very little to very much trust, 90% of the population placed high or very high trust in general practitioners (GPs) and medical specialists. Dentists, pharmacists and nurses are highly or very highly trusted by 80 to 90% of the population [10].

Complementary and alternative medicine (CAM) is in the Cochrane Complementary Medicine Field [11] defined as including: "all such practices and ideas which are outside the domain of conventional medicine in several countries and defined by its users as preventing or treating illness, or promoting health and well-being". In the Netherlands, public trust ratings in this type of medicine are expected to be lower.

Firstly, rating of public trust in CAM might be lower, because high levels of public trust in conventional medicine might be a result of institutions that implicitly guarantee trust, in the case of health care warranting the expectation of a certain level of quality of care [2]. A guarantee of trust is, for instance, that only certified care providers are allowed to work in health care. Another guarantee is that patients, who are visiting a caregiver in conventional medicine, are treated by providers with a special education often perceived to be working according to protocols based on best available evidence. Such guarantees might lead to higher levels of trust, especially public trust in conventional medicine. In the broad range of CAM philosophies and therapies, some of the above mentioned institutional guarantees are not apparent or less convincing as CAM providers also include those who are not certified and whose educational requirements are unclear. In addition, unlike conventional medicine CAM practices are thought to lack reliable, scientifically based information. The absence of these types of institutional guarantees will possibly affect the amount of public trust in CAM.

Secondly, trust in CAM might be lower than trust in conventional health care and health care providers as it is partly build up through experience. Less people have experience with CAM than with conventional medicine. The effect might be lower levels of trust, as has previously been demonstrated with regard to trust in mental health care providers [12].

Moreover, in the Netherlands CAM has been subject of public debate, much more so than conventional medicine. A case in point was the death of a Dutch media personality in 2001 that refrained from breast cancer treatment and relied on CAM healers [13]. The ensuing negative media attention might have affected the level of public trust in CAM.

However, it is not known what the level of public trust of CAM is in the Netherlands and what factors actually influence the level of public trust in CAM. The aim of this study is to gain insight into public trust in CAM and the determinants that lie at the root of it. We will elaborate on this aim by answering two questions. The first, descriptive, question deals with the level of public trust in CAM:

- How much trust does a representative sample of the Dutch population place in CAM in general and in a number of specific types of CAM?

We will elaborate on potential influencing factors in the next section, when we formulate hypotheses answering our second explanatory question:

- Which determinants influence the level of public trust in CAM?

In contrast with conventional medicine, not all Dutch inhabitants have experiences in the field of CAM. Therefore, with regard to both questions, a division will be made in those who have used and those who have not used CAM.

### Hypotheses

In a model we developed previously [14,15], we distinguished four groups of influences on public trust in conventional medicine:

1. Institutional guarantees

2. Media exposure

3. Network knowledge

4. Personal experiences with the health care system.

Regarding public trust in CAM we adapted these groups of influencing factors to refer to CAM. We also added two other influences that might be especially relevant in the case of CAM:

5. The advice of providers of conventional medicine, notably GPs

6. Public trust in conventional medicine.

**Figure 1:**
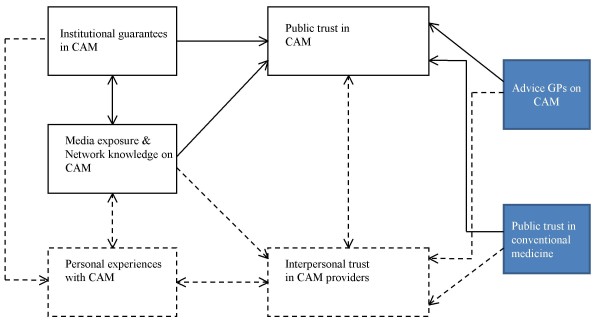
**Model of public trust in CAM medicine**.

Figure 1 displays the adjusted model for public trust in CAM.

For CAM users, all groups of influences are applicable. For CAM non users, the groups of influences in the dotted boxes in Figure 1 (personal experiences and interpersonal trust in CAM providers) and the dotted lines do not play a role in placing public trust in CAM.

#### Institutional guarantees

In general, one of the determinants of public trust is formed by social institutions that reduce the risk of placing trust in someone or an organisation, viz. institutional guarantees. These guarantees relate to basic conditions, such as government regulation of education of health care providers, protection of patients' rights and independent reviews of health care quality. Some of these conditions are based on legal regulations, whilst others are based on the self-regulation of the professions. Examples of the latter are membership and quality regulation by professional associations or voluntary quality certification. Research on the use of CAM by chronically ill people [16] concluded that health policy makers should be alert to the quality of CAM health providers, for instance by insisting on professional certification. Concerning CAM, it is hypothesized that these institutional guarantees will increase the levels of public trust in CAM and their providers.

H: The presence of institutional guarantees will lead to more people placing trust in CAM.

#### Media exposure

According to our trust model, public trust in general and thus in CAM is also influenced by media [17]. Health and health care are popular subjects in the mass media. We expect that exposure to positive (or negative) media information on CAM will influence public trust in a positive (or negative) way. Mass media information about therapies pays a lot of attention to CAM therapies [18,19]. A review on media coverage on CAM [20] indicates that for the most part this appears to be positive. The majority of CAM reporting in newspapers in the UK and Germany is positive [21]. In the US, UK, China, Japan and Israel coverage was found to be overwhelmingly positive with 58% of the articles containing some positive portrayal or support of CAM, while only 20% contained negative portrayal [22]). Also, in a study on 16 years of Canadian newspaper and magazine coverage, a larger proportion of articles was judged to be favourable towards CAM use for cancer than not (61.3% of magazine and 45.3% of newspaper articles) [23]. The literature indicates an overall positive tendency on media on CAM. However, this might differ between countries. In Germany for instance reporting may be more critical than in the UK [21]. In the Netherlands in 2001, as was mentioned in the introduction, there was negative media hype on CAM. Consequently the media might have had an influence on public trust in CAM especially in the Netherlands at that time.

H: People who mainly were exposed to positive media information on CAM will be more trusting toward CAM than people who mainly were exposed to negative, neutral or no media information on CAM.

#### Network knowledge

Social networks in general influence trust in two ways: by providing information on the trustworthiness of others and by providing instruments to sanction betrayal of trust [24]. Important others in one's network might provide clues on whether or not to place trust in CAM. No research is available on social networks in relation to public trust in CAM. However, several studies have been conducted on the role of networks during the decision making process for using CAM. These studies show that people use their network in deciding to use CAM. One study [25] on trusted information sources regarding CAM showed that users of CAM modalities obtained substantial amounts of information from family, friends and co-workers. Although this information was not rated high by these users, these interpersonal contacts informed them about experiences of other CAM users, giving the potential user a personal view on the possible effectiveness of the modality and providing them with the opportunity to ask questions and judge the modality on its trustworthiness. A study on the influence of significant others in CAM decisions by Canadian cancer patients has shown that multiple types of decisional involvement by significant others were identified: creating a safe place for the patient to make a decision, collaborative decision making, moving the patient towards a decision and making the decision for the patient [26]. In this study it was found that although partners did comprise the majority of significant others as being involved in CAM decisions, friends and other family members, such as adult children and parents, also played an important role.

Different patients may appreciate different forms of information when making decisions regarding their health [27]. As a consequence for some patients family, friends or close associates who offered opinions and personal testimonials often initiated treatment suggestions whereas for others family and friends acted as information conduits only.

These studies might imply that next to the influence of networks on the decision making process to use CAM, information provided through networks might also, to some extent, have an impact on the amount of public trust placed in CAM. When network members share their positive experiences with CAM, this information lowers the risk one takes when placing trust in CAM, increasing the amount of trust in CAM. A negative experience of network members might lead to lower levels of trust in CAM compared to people who did receive positive information or people who were not informed by their network.

H: People who received mainly positive information on CAM from their network will be more trusting toward CAM than people who received mainly negative, neutral or no information on CAM.

#### Personal experiences with the health care system

Previous experiences with CAM might play an important role in placing public trust in CAM. Previous experience with the *same *health care provider confers information on the trustworthiness of this specific provider. This interpersonal trust, rooted in dyadic embeddedness [24], might be generalized to other providers or the system as a whole. We therefore expect that previous CAM users will place more trust in CAM modalities, if their experiences were positive. Positive experiences may increase public trust in CAM over time. A serious failure will possibly affect public trust in CAM in a negative manner.

Most Dutch visitors of CAM practitioners are positive about the provided treatment and information given by the CAM provider [28]. A study conducted in the UK also found that only very few patients made negative comments about their experiences with complementary therapies [29]. This might imply that most of the CAM users are positive regarding their treatment, leading to more public trust in CAM.

H: Personal experience with CAM is expected to increase trust in CAM and for those having personal experience people who had mainly positive experiences with CAM will be more trusting toward CAM than people who had mainly negative, neutral or no experiences.

#### The advice of providers of conventional medicine, notably GPs

The advice of a provider of conventional medicine concerning CAM might influence the level of public trust in CAM. The assumption is that if providers of conventional medicine inform their patients in a positive way on CAM, they will be more willing to place trust in CAM. GPs have a status of personal and highly trusted [10] doctors. Robinson et al [26] found that both CAM non users as well as users highly trusted their doctors and rated their information at the highest level. Information obtained from doctors, such as one's GP, may feel as reliable, scientifically based information, as GPs are expected to have expertise in scientifically based health care [26]. This might lead to their patients taking the information into serious consideration. This is especially the case in health care systems with a gate-keeping system, such as the Netherlands. Evidence about participation in cervix screening illustrates the influence of GPs: invitation and reminder by women's own GP increased participation [30].

Therefore, it is expected that for those who received positive information from their GP about CAM this might lead to higher levels of trust.

H: People who received mainly positive information on CAM from providers of conventional medicine will be more trusting toward CAM than people who received mainly negative, neutral or no information on CAM.

#### Public trust in conventional medicine

Low levels of public trust in conventional medicine are expected to be related to higher levels of public trust in CAM. Studies on reasons for using CAM do not provide a clear picture. Lee Treweek [31] argues that one of the reasons for a rise of the use of CAM is dissatisfaction with both medical encounters and outcomes. However, there is also evidence showing that CAM therapies are used alongside conventional medicine [32] and that CAM use is not related to dissatisfaction with conventional care [33]. Sirois [34] found a shift in motivations to use CAM towards motivations focussing more on the positive aspects of CAM and less on the negative aspects of conventional medicine. Van den Brink-Muinen and Rijken [16] showed that the use of CAM in chronically ill people was related to lower trust in conventional medicine professions and higher trust in CAM practitioners. This suggests that there might be a negative relation between trust in conventional medicine providers and trust in CAM providers.

H: People who place less trust in conventional medicine will be more trusting toward CAM.

## Methods

### Sample

The sample comprises respondents of the "Dutch Health Care Consumer Panel". This panel consists of approximately 1500 members and is a cross-section of the Dutch population. One third of the Consumer Panel is renewed every two years. This renewal ensures that the panel remains a cross-section of the population, that members do not develop specific knowledge of and attention for health care issues and no "questionnaire-fatigue" occurs. New members for the panel are sampled from the general population. Sampled people receive an information letter about the panel and are called within a week after receiving that letter. If they are interested they receive a questionnaire on background characteristics. When that questionnaire is returned they are considered members of the panel. The panel is registered by the Dutch Data Protection Authority (no. 1262949).

The data of the present study were gathered in December 2001 by a postal questionnaire. At that time the panel consisted of 1358 members. 917 postal questionnaires were returned, which equals a response rate of 65%.

### Questionnaire

The "public trust in CAM" questionnaire was based on a validated "public trust in health care questionnaire" [8]. In the Netherlands, instead of CAM usually the term alternative medicine is used to indicate this type of care. In the questionnaire, we therefore asked about public trust in alternative medicine. However, while both alternative medicine and CAM refer to the same sort of therapies, in this article the international term CAM is used.

Respondents were asked to mark their level of trust in CAM in general, ranging from 1 (no trust at all) to 10 (complete trust). Next to that overall mark on the broad field of CAM, respondents could indicate their level of trust in specific CAM therapies, including acupuncture, homeopathy, manual therapy, paranormal therapy, naturopathic therapy and anthroposophy. This was measured, unlike public trust in CAM in general, on a 4-point-scale (1 = very little, 2 = little, 3 = much and 4 = very much).

To gain information on institutional guarantees respondents could point out to what extent their trust would decrease or increase when:

- a CAM practitioner is a member of a professional association;

- a CAM practitioner has a quality certification mark;

- a CAM practitioner has a degree in CAM;

- a CAM practitioner has a degree in conventional medicine.

The answering categories of the questions on institutional guarantees were: decreases a lot, decreases, no increase/no decrease, increases and increases a lot.

With regard to media exposure, respondents could indicate if they had read or heard about CAM in the media, and if they perceived the information as positive, neutral or negative (1 = very negative, 2 = negative, 3 = neutral, 4 = positive, 5 = very positive).

Network information on CAM was measured, firstly, by a question on whether they received any information on experiences with CAM by their friends, family or acquaintances. If the respondents had received information, they could mark whether it was, to their opinion, positive, neutral or negative information (1 = very negative, 2 = negative, 3 = neutral, 4 = positive, 5 = very positive).

On CAM use, two questions were asked. Firstly, the respondents were asked whether they had used CAM products. Secondly, they could report if they had attended a practitioner in CAM. Answering categories of both questions were 'yes, at this moment', 'yes, in the past' or 'no'. If they had used products or attended a CAM practitioner, the respondents could point out if they perceived this as positive, neutral or negative (1 = very negative, 2 = negative, 3 = neutral, 4 = positive, 5 = very positive).

Advice of a health care provider on alternative medicine was measured by a question on whether their GP had advised them on CAM. If they were advised, respondents were asked to describe how they had perceived the nature of this information on a 5-point-scale (1 = very negative, 2 = negative, 3 = neutral, 4 = positive, 5 = very positive).

Public trust in conventional medicine was measured by one item, as was public trust in CAM. Respondents could mark their level of public trust, ranging from 1 (no trust at all) to 10 (complete trust).

### Data analysis

To indicate whether a person, to his/her perception, had received positive, neutral, negative or no information from their network, the media or their GP the two questions were recoded into one variable. In the new variable the respondents who had answered that they did not receive information from a source were placed in the 'no information' category. If the respondents were informed on CAM by the source and had reported that the content of the information was negative, neutral or positive, in the new variable they were placed in the matching category with the values: negative, neutral or positive information. This newly constructed variable consisted of 4 answering categories, namely 'no information', 'negative information', 'neutral information' or 'positive information'. In the same way, a new variable was constructed on experiences with CAM. Respondents who did not have experience with the usage of products and visits to therapists were gathered in the group 'no experience'. Respondents who had experience at this moment or in the past with products or visits were classified as negative, neutral or positive experienced.

After recoding the variables analyses to explore the relation to public trust in CAM were performed. Firstly, trust placed in institutional guarantees was analysed by frequency tables. Then these items were combined to form a scale for latter analyses. Secondly, to study whether experiences, network, media exposure or trust in conventional medicine were related to the level of trust ANOVA and post hoc tests were performed. Finally, a linear regression analysis (pairwise deletion) was conducted with overall trust in CAM as the dependent variable and institutional guarantees (scale), network, media exposure, experiences and trust in conventional medicine as independent variables. Therefore, nominal variables were recoded into dummy variables. This analysis was controlled for age, sex and education. These analyses were performed for the total sample and, as personal experience with CAM products or providers seemed to be important determinants of public trust in CAM, separately for those with and without personal experience with CAM. SPSS 11.5 was used.

## Results

### Sample characteristics

In the total sample, the mean age of the 917 responding panel members was 53.1 (SD 14.7). 48.4% were male and 51.8% were female respondents. Most of them had completed low (43.7%) or medium education (33.3%), 23.0% was highly educated (higher vocational training/university). 74.6% of the respondents perceived their health status to be good or excellent, 23.4% average and 2.0% poor. 40.7% of the population indicated themselves as CAM users. Of the CAM users, 34.2% used CAM at this moment, whereas 65.8% pointed out to have used CAM in the past. 47.6% of the current users combined the usage of CAM products with visits to a CAM practitioner, 41.5% only used products and 10.9% only visited a practitioner. More than half of the past users (53.1%) used both products and visited a CAM practitioner, 23.8% solely used CAM products and 23.1% solely went to see a practitioner. CAM users were more often female (Chi^2 ^= 32.22; df = 1, p < .000) and slightly younger than non users (49.7 versus 53.9) (F = 13.47; df = 790; p < .000). Also, they were slightly higher educated (Chi^2 ^= 7.08; df = 2; p < .05). No differences were found between CAM non users and users in their perceived health status.

### Public trust in CAM

**Figure 2:**
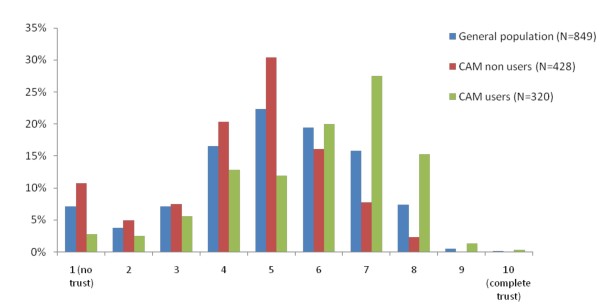
**Degree of public trust in CAM on a 1 to 10 scale**.

The total sample judged trust in CAM with an average grade of 5.05 (SD 1.88). CAM users were significantly more trusting toward CAM with an average of 5.85 (SD 1.75) than those did not use CAM (F:.86; df 746; p < 0.001). Their average trust judgement of CAM was 4.46 (SD 1.81). Figure 2 shows the distribution of public trust in CAM for the total sample, CAM users and non users.

Of the specific types of CAM, asked in the questionnaire, manual therapy, homeopathy and acupuncture are trusted most by the total sample, CAM users and CAM non users (Table 1). Paranormal therapy is the least trusted of CAM therapies. CAM users place more trust in the presented therapies than CAM non users. The difference is smallest for acupuncture, relatively small for manual therapy and homeopathy, and bigger for naturopathic therapy and anthroposophy. The biggest difference between CAM users and non users is found for paranormal therapy.

**Table 1:** Trust in specific types of CAM therapies

	Total sample		CAM non users		CAM users	
	
	Percentage much/very much trust	N*	Percentage much/very much trust	N*	Percentage much/very much trust	N*
Manual therapy	54.3%	736	37.9%	354	74.6%	295
Homeopathy	49.1%	751	32.7%	355	66.4%	307
Acupuncture	48.3%	719	38.6%	350	59.4%	286
Naturopathic therapy	22.3%	660	12.2%	320	34.6%	263
Anthroposophy	19.4%	578	9.7%	289	30.4%	214
Paranormal therapy	10.0%	703	4.2%	337	17.1%	281

* People who had 'no opinion' on trust in this type of alternative medicine were not taken into account in the N.

### Determinants of public trust in CAM

#### Institutional guarantees

In the total sample, membership of a professional association, a quality certification mark and a degree in CAM leads for around 50 to 60% of the respondents to an increase in trust in a CAM practitioner (Table 2). Trust increases most when a CAM practitioner has had a degree in conventional medicine; 86.3% of the population responded that if this was the case their trust would increase. For all four institutional guarantees CAM users displayed higher levels of increasing trust than non users. The difference is smallest, however, in case of a degree in conventional medicine.

**Table 2:** Institutional guarantees and trust in CAM

	Professional association	Quality certification mark	Degree in CAM	Degree in conventional medicine
**Total sample**	(N = 871)	(N = 871)	(N = 872)	(N = 870)
Highly increases trust	6.0%	5.3%	5.7%	27.9%
Increases trust	53.0%	48.7%	47.0%	58.4%
Neutral	39.3%	44.0%	45.6%	12.0%
Decreases trust	0.5%	0.7%	0.3%	0.6%
Highly decreases trust	1.3%	1.4%	1.3%	1.1%
**Total**	100%	100%	100%	100%

**CAM non users**	(N = 447)	(N = 446)	(N = 447)	(N = 446)
Highly increases trust	2.0%	2.2%	2.0%	21.5%
Increases trust	45.9%	43.3%	40.5%	61.4%
Neutral	49.0%	51.1%	54.8%	13.9%
Decreases trust	0.9%	1.1%	0.7%	0.9%
Highly decreases trust	2.2%	2.2%	2.0%	2.2%
**Total**	100%	100%	100%	100%

**CAM users**	(N = 324)	(N = 324)	(N = 324)	(N = 325)
Highly increases trust	11.1%	9.6%	10.8%	36.0%
Increases trust	61.4%	54.0%	53.7%	53.8%
Neutral	27.2%	35.5%	34.9%	9.8%
Decreases trust	-	0.3%	-	0.3%
Highly decreases trust	0.3%	0.6%	0.6%	-
**Total**	100%	100%	100%	100%

#### Media exposure

Of those in the total sample who were informed by the media, 13.0% indicated that they were exposed to positive information (Table 3). 38.1% of the respondents reported that they had been exposed to negative media information. About one fifth of the respondents (17.3%) did not report information on CAM through the mass media. In both CAM non users and users, most respondents indicated to be informed negatively (36.9% and 41.2%). In the total sample, information received from the media is significantly associated with trust ratings on CAM. People who reported to be positively informed by the media were significantly more trusting toward CAM than those who perceived the media information to be negative. This association was also found for CAM non users, not for CAM users.

**Table 3:** Availability of information and trust in CAM

	%	Total sample Mean trust in CAM at present (SD)	%	CAM non users Mean trust in CAM at present (SD)	%	CAM users Mean trust in CAM at present (SD)
Media exposure	(N = 892)	(N = 839)	(N = 463)	(N = 423)	(N = 323)	(N = 318)
Positive information media	13.0%	*6.00 (1.71)	10.6%	*5.73 (1.59)	16.7%	6.29 (1.78)
Neutral information media	31.6%	*5.18 (1.62)	31.5%	*4.68 (1.40)	29.1%	5.82 (1.71)
Negative information media	38.1%	*4.61 (1.93)	36.9%	*3.84 (1.62)	41.2%	**5.60 (1.86)
No information media	17.3%	*5.20 (2.03)	21.0%	*4.60 (2.10)	13.0%	6.29 (1.64)
						
Network knowledge	(N = 893)	(N = 838)	(N = 465)	(N = 424)	(N = 321)	(N = 316)
Positive information network	33.6%	*6.22 (1.58)	16.8%	*5.39 (1.62)	59.5%	*6.56 (1.52)
Neutral information network	12.1%	*4.63 (1.43)	12.5%	*4.45 (1.29)	10.6%	*4.94 (1.46)
Negative information network	10.8%	*3.43 (1.56)	11.2%	*3.24 (1.47)	9.7%	*3.69 (1.58)
No information network	43.6%	*4.63 (1.76)	59.6%	*4.42 (1.78)	20.2%	*5.28 (1.65)
						
Experience	(N = 798)	(N = 748)	(N = 428)	(428)	(N = 320)	(N = 320)
Positive experience	27.7%	*6.56 (1.39)		-	68.0%	*6.56 (1.39)
Neutral experience	6.4%	*5.12 (1.49)		-	15.7%	*5.12 (1.49)
Negative experience	6.6%	*3.54 (1.48)		-	16.3%	*3.54 (1.48)
No experience	59.3%	*4.46 (1.75)	100%	4.46 (1.75)	-	-
						
Advice GP	(898)	(N = 842)	(N = 466)	(N = 424)	(N = 323)	(N = 318)
Positive information GP	7.2%	*6.35 (1.47)	2.1%	*5.70 (1.34)	14.9%	*6.63 (1.50)
Neutral information GP	6.5%	5.49 (2.09)	2.4%	*3.80 (2.53)	13.9%	5.93 (1.80)
Negative information GP	2.4%	*5.36 (2.50)	1.3%	*4.00 (2.97)	4.6%	5.80 (2.24)
No information GP	83.9%	*4.90 (1.82)	94.2%	4.45 (1.70)	66.6%	5.67 (1.81)
						
Trust convent. medicine	(N = 761)	(N = 707)	(N = 385)	(N= 353)	(N = 274)	(N = 269)
Lot of trust (7-10)	68.3%	5.14 (1.88)	71.2%	4.59 (1.73)	62.8%	6.01 (1.77)
Not a lot/not a little trust (5-6)	29.5%	5.01 (1.89)	26.8%	4.29 (1.62)	35.0%	5.73 (1.93)
Little trust (1-4)	2.2%	4.20 (2.04)	2.1%	3.63 (2.07)	2.2%	5.00 (2.35)

* ANOVA post hoc test, p < 0.001

#### Network knowledge

In the total sample, 33.6% perceived the information from their network as positive, as opposed to 10.8% who learned through their network about negative experiences with CAM. 43.6% did not receive information from family, friends or acquaintances on experiences with CAM. In the CAM non users population almost 60% indicated that they were not informed on CAM by their network, whereas almost 60% of the CAM users reported to be positively informed through this source.

In the total sample, positive information on CAM as reported by their network is significantly associated with a higher trust level, negative information with significantly lower levels. Although CAM non users display lower levels of trust than CAM users, the same association is found for both CAM non users and CAM users.

#### Personal experiences with the health care system

As was mentioned earlier, of the total sample, 40.7% had had experience with CAM in their life time; of those 27.7% reported a positive, 6.4% a neutral and 6.6% a negative experience. Almost 60% did not have experience with CAM. Experience was associated with public trust in CAM. People who had had neutral or negative experiences were significantly less trusting toward CAM.

#### The advice of providers of conventional medicine, notably GPs

In the total sample, 7.2% perceived information from their GP as positive and 6.5% as neutral; 2.4% reported that the information from their GP was negative. Over 80% of respondents indicated that they did not receive any information from their GP on CAM. CAM users seem to discuss their use more often with their GP in contrast with non users. 33.4% of the CAM users reported to have received information from their GP as compared to 5.8% of the non users.

In the total sample, positive information received from GPs is associated with higher levels of trust concerning CAM rather than negative or no information. Positive information is also associated with higher levels of trust among both CAM non users and users.

#### Public trust in conventional medicine

In all three groups, conventional medicine is highly trusted by most respondents. No relationship between public trust in conventional medicine and public trust in CAM was found in the general, CAM non using and using population.

#### Regressions

The regression analysis of the total sample shows that 41% of the variance in overall trust in CAM is explained by the variables used (Table 4). Overall trust was related to people's belief in the role of institutional guarantees: when they ascribed a trust enhancing role to professional associations, quality certification, a degree in CAM and a degree in conventional medicine, their overall trust in CAM was higher.

**Table 4:** Regression public trust in CAM at present, total sample, CAM non users, CAM users

	Total sample		CAM non users		CAM users	
	R^**2 **^adj = .41		R^**2 **^adj = .31		R^**2 **^adj = .48	
	
Model	B	sign	B	sign	B	Sign
Constant	.141	.827	-.513	.579	2.684	**.004**
						
Institutional guarantees	**1.153**	**.000**	**1.179**	**.000**	**.738**	**.000**
						
Pos vs no info media	-.030	.892	.591	.058	-.288	.357
Neutral vs no info media	-.221	.198	.017	.939	**-.637**	**.021**
Neg vs no info media	**-.734**	**.000**	**-.635**	**.004**	**-.611**	**.023**
						
Pos vs no info network	**.746**	**.000**	.395	.092	**.710**	**.001**
Neutral vs no info network	-.225	.228	-.090	.714	-.086	.788
Neg vs no info network	**-1.084**	**.000**	**-.934**	**.000**	-.257	.461
						
Experience vs no experience	**.674**	**.000**				
						
Pos vs neutral experience					**1.086**	**.000**
Neg vs neutral experience					**-1.018**	**.000**
						
Pos vs no info GP	.267	.249	-.067	.905	.255	.285
Neutral vs no info GP	.132	.580	-.129	.804	.111	.643
Neg vs no info GP	-.029	.939	-.077	.911	.126	.748
						
Trust regular h. care	.090	.127	.156	.067	.013	.873
						
Female vs male	-.019	.707	.048	.555	-.021	.759
						
Age	-.001	.860	-.003	.634	-.006	.338
						
Level of education	.002	.521	.003	.440	-.002	.636

People who perceived the information from the media as negative were significantly less trusting toward CAM as opposed to people who had not seen or heard information on CAM in the media. Trust-judgements of those who reported positive or neutral media information were not significantly affected, compared to people who did not receive media information.

People who reported to be positively or negatively informed by their network were significantly influenced in comparison to people who did not receive information through this source.

Experiences also play a significant role. In comparison to people who did not report any experience with CAM, trust levels of those who had experiences were significantly higher.

Information from the GP, trust in conventional medicine, sex, age and educational level did not significantly relate to the level of public trust in CAM.

The separate regression analyses for CAM non users and users show that public trust in CAM is significantly related to institutional guarantees, although more prominent for the CAM non users (Table 4). Perceived negative information from the media coincides for both CAM non users and users with lower levels of public trust in CAM. For the users, also neutral information has a negative relationship. CAM users are significantly influenced by perceived positive information from their network, having a higher level of trust. In contrast, trust-judgements of non users are negatively related to perceived negative information from their network. Both positive and negative experiences have an impact on their trust judgements. In both groups, information from the GP, trust in conventional medicine, sex, age and educational level did not affect the level of public trust in CAM.

## Discussion

This study aimed to provide insight into the level of public trust in CAM and the determinants which influence this trust in the Dutch population. The level of public trust in CAM was 5.05 on average on a scale of 1-10. People who had personal experience with CAM products or providers were significantly more trusting. As hypothesized, public trust in CAM in the Netherlands was associated with several conditions. Firstly, institutional guarantees play an important role in placing trust in CAM. People indicated that their trust would be higher when institutional guarantees were apparent. Another indication is that the three, in our study, most trusted types of CAM (manual therapy, homeopathy and acupuncture) are usually provided by medical doctors or physiotherapists. In the regression analysis, public trust in CAM was associated with institutional guarantees, negative media information, positive and negative information reported by their social network and people's personal experiences with CAM. The regression analyses on CAM non users and users display that the emphasis on what relates to public trust in CAM differs between both groups. For CAM non users, trust is mostly associated with institutional guarantees, whereas for CAM users their experiences, positive or negative, are most important. Institutional guarantees also play a role for CAM users, although to a lesser extent. For both non users and users, trust levels in CAM are affected by negative media information. Trust levels of CAM users are also related to neutral media information. Public trust in CAM amongst non users is related to negative information from their network; public trust in CAM amongst CAM users is significantly related to positive information from their network.

The analyses presented in this article confirm to a large extent the hypotheses that were part of our basic model of public trust in health care: institutional guarantees, media exposure, network knowledge and personal experience (through interpersonal trust) influence public trust in CAM.

However, next to the fact that our basic model is largely confirmed, the two hypotheses we added especially for their supposed relevance to CAM were refuted. The lack of a relationship between advice by one's GP and public trust in CAM might be due to the fact that only 16% of the respondents reported to have received advice on CAM from their GP. Not surprisingly, the lowest rate on having had advice on CAM were found in the CAM non users group (5.8%). However, also amongst CAM users the percentage of those receiving advice on CAM through their practitioner was relatively low (33.4%). This relatively small part of the population discussing CAM with their GP might implicate that people feel uncomfortable sharing information on CAM with their GP. Support for this statement is found in several other studies. A review by Robinson and McGrail [35] of 12 studies into patient communication of CAM use to their medical practitioner found non-disclosure rates as high as 72%. Three main reasons for non-disclosure of CAM use were found. The first and most common reason is concern about a negative reaction from the medical practitioner [35,36], characterizing the patient as "fringe, ungrateful, unrealistic, or gullible" [37]. The second reason is the patient's perception that the medical practitioner did not need to know [35], because CAM modalities used were irrelevant to the biomedical treatment course [36,37]. The third reason is that the medical practitioner appeared disinterested or did not ask [35,36].

The hypothesized negative relationship between public trust in conventional medicine and public trust in CAM was also not found. Instead, there is rather a very weak tendency towards a positive relationship between public trust in CAM and in conventional medicine. However, the basis for these correlations is quite different: higher levels of trust in conventional medicine and lower levels of trust in CAM among non CAM users, and lower levels of trust in conventional medicine and higher levels of trust in CAM among CAM users. The tendency toward a positive relationship between public trust in conventional medicine and CAM might reflect an underlying tendency to be trustful towards different objects of trust.

The differences in the levels of public trust in CAM and the determinants that lie at the root of it between CAM users and non users provide an insight in placing trust in CAM. CAM users display a significantly higher level of trust in CAM than non users. Firstly, this can be explained by their experiences. Experiences, positive or negative, are significantly related to placing public trust in CAM. Most of the users reported positive experiences (68.1% of 40.7% who reported experience with CAM), leading to a higher level of public trust. Secondly, this study showed that CAM users perceived significantly more positive information from their network on CAM than those who did not use CAM. This implicates that CAM users possibly relate to those who have a positive attitude towards CAM. This positive information might also lead to higher levels of trust. CAM non users might not surround themselves with people with a positive perception on CAM. It was found that most non users did not receive any information through their network. However, for those who did receive negative information from their network, this had a negative impact. Thirdly, this study also showed that CAM users reported somewhat more often that they had received CAM information through mass media, compared to CAM non users. However, this attention effect did apparently not coincide with selective perception or confirmation bias of media information as positive or negative. In contrast with the network surrounding a person, if media displays negative information CAM users and non users take it up and this exposure to negative information has an impact on public trust in CAM in both groups.

This study is the first to provide an insight in public trust in CAM and its determinants. However, it has some limitations. A first limitation is that it relies solely on self-reported information of the respondents. People were asked to report about their experience with CAM and whether or not the media and their network informed them positively or negatively on CAM. This self-reported information might be biased. People might tend to consistency in their answers in the same questionnaire ('same-source bias'). By the same token, people consciously or unconsciously tend to search for and interpret information in a way that confirms their existing ideas, leading to what is called 'confirmation bias' [38]. As a result of this, we might have overestimated the influence of institutional guarantees and information on public trust in CAM. However, concerning the mass media, we did find selective take up in the sense of reporting having had information from the media, but no confirmation bias.

A second limitation is that our definition of experience with CAM was fairly liberal. In our study infrequent, new users of CAM and established CAM users were treated as a homogenous group, whereas research indicates that these are distinct groups, differing on reasons for using CAM as well as on treatment patterns. Sirois and Gick [39] found that for initial/infrequent users, the best predictors of using CAM were dissatisfaction with conventional medicine and health aware behaviors, while more frequent health aware behaviors were associated with continued CAM use. Medical need also influenced the choice of using CAM. It was the best predictor of committed CAM use, with established CAM clients reporting more health problems than the initial/infrequent CAM group. With regard to treatment patterns, Sirois [40] found that newer CAM users still depend greatly on conventional medicine, whereas more experienced CAM users rely less on conventional medicine alone and more on CAM for treatment of their non-life-threatening health issues. If in our study a distinction could be made between initial/infrequent users and established users of CAM, some of the results of our study might differ per group. For example, established CAM users would be expected to be more trusting compared to initial/infrequent users.

A third limitation is that we used a broad measure of public trust in CAM as dependent variable in the analyses. However, it might be that a person trusts one type of CAM therapy, but not the other. In our analyses, we did not distinguish between trust in types of CAM therapies.

Another limitation is that the data were collected in 2001, which leads to the question whether the study results are still applicable to the current Dutch situation. There is reason to believe that changes in public opinion toward CAM and experience with CAM within a population might lead to more positive attitude and possibly higher trust ratings toward CAM. A study [33], mentioned earlier in the introduction, on a Canadian population found that in an eight year period of time increasing usage led to a shift in motivations focusing more on the positive aspects of CAM and less on the negative aspect of conventional medicine. If changes in CAM use were also found in the Dutch population, this might have affected trust ratings. However, usage of CAM, in terms of visiting a CAM practitioner, remained stable from 2001 until 2008 (between 6-7%) [41]. There are no data available about a change in the use of CAM products. Increasing usage might not have affected public knowledge and opinion with regard to public trust in CAM. What might have affected the trust ratings are the media. In 2001, the media might have been more negative towards CAM, with regard to the incident reported in the introduction. 38.1% reported that the media information they were exposed to was negative. This might have affected the level of trust to some extent and possibly led to an overestimation of the reporting and impact of negative media information. However, changes in public trust in CAM will be small due to the relatively stable character of trust. A longitudinal study on changes of public trust in conventional medicine showed that public trust in conventional medicine remains at a constant level [14].

The last limitation is that it is unclear to what extent the study results can be generalized to other countries. In our opinion, our basic model is general and applicable in other countries. However, the level of trust and strength of the impact of the determinants of public trust in CAM depend on the position of CAM within the care system and the culture of a country. For example: institutional guarantees might differ from one country to another, but its relationship to public trust in CAM might be the same. In the same way media coverage of CAM might differ. Such differences between countries might influence the level of trust in CAM, but the determinants may still have a comparable impact on public trust in CAM.

Therefore, differences in public trust in CAM and the impact of determinants should be studied in different countries. Moreover, future research should pay attention to changes in public trust in CAM over time and concomitant changes in the impact of determinants.

## Conclusions

The level of public trust in CAM in the Dutch population was 5.05 on average on a scale of 1-10, much lower than trust in regular health care. CAM users were significantly more trusting toward CAM than non users. With regard to the hypotheses, it can be concluded that those on institutional guarantees, media exposure, information from people's social network and their personal experiences are largely confirmed by our study for the total sample, CAM non users and users. The hypotheses about the influence of GPs and about the relationship between trust in CAM and trust in conventional medicine were rejected.

## Competing interests

The authors declare that they have no competing interests.

## Authors' contributions

ES was responsible for gathering the data, performing the statistical analysis, interpreting the data and drafting the manuscript. PPG was involved in interpreting the results and in revising the manuscript critically for important intellectual content. Both authors read and approved the final manuscript.

## Pre-publication history

The pre-publication history for this paper can be accessed here:

http://www.biomedcentral.com/1471-2458/10/128/prepub
